# Forensic psychiatric patients’ experiences of participating in administrative court proceedings concerning the continuation of forensic psychiatric care

**DOI:** 10.3389/fpsyt.2023.1151554

**Published:** 2023-03-16

**Authors:** Andreas Söderberg, Märta Wallinius, Christian Munthe, Mikael Rask, Ulrica Hörberg

**Affiliations:** ^1^Department of Health and Caring Sciences, Linnaeus University, Växjö, Sweden; ^2^Centre for Ethics, Law and Mental Health, Department of Psychiatry and Neurochemistry, Institute of Neuroscience and Physiology, The Sahlgrenska Academy, University of Gothenburg, Gothenburg, Sweden; ^3^Research Department, Regional Forensic Psychiatric Clinic, Växjö, Sweden; ^4^Department of Clinical Sciences Lund, Child and Adolescent Psychiatry Research Unit, Lund University, Lund, Sweden; ^5^Department of Philosophy, Linguistics and Theory of Science, University of Gothenburg, Gothenburg, Sweden

**Keywords:** administrative court proceeding, forensic psychiatry, patient perspective, phenomenology, psychiatry

## Abstract

**Introduction:**

Previous studies show that both staff and patients describe patient participation as a challenge in forensic psychiatry. One reason may be that the forensic psychiatric process is difficult to understand and is experienced as being slow and complex. The proceedings in an administrative court are a core element in forensic psychiatric care as it constitutes the legal authority that legitimizes the deprivation of liberty. A better understanding about how patients experience these proceedings can contribute with important knowledge about how forensic psychiatric care can be understood from a patient perspective. The aim of the study was to describe patients’ lived experiences of participating in oral hearings in an administrative court concerning the continuation of their forensic psychiatric care.

**Materials and methods:**

This is a phenomenological study performed in a Swedish context with a total of 20 interviews conducted with a Reflective Lifeworld Research (RLR) approach.

**Results:**

The results reveal three themes; A significant, correct but meaningless formality; An imbalance of power within the hearings; and Existential and practical disorientation.

**Conclusion:**

The findings show how these court proceedings concerning the continuation of forensic psychiatric care are often experienced as challenging. This is partly due to the care structure in forensic psychiatry and that the purpose of the hearings is difficult to comprehend and is perceived as unjust by patients. Another challenge is of a more existential dimension, where the main character in a hearing is most likely in a situation that would be stressful for anyone. However, the focus on danger can make this experience even more intense. An increased transparency on this legal process along with more discussion and education for both patients and staff is called for based on the results.

## 1. Introduction

Sweden has a specific mental health legislation for criminal offenders with *severe mental disorders*^1^ (2). This legislation differs from that in most other countries since it does not have a legal insanity defense, but a presumption against sentencing convicted severely mentally disordered offenders to prison. Such offenders are instead held legally accountable for their crimes but are sentenced to forensic psychiatric care, which is a *bona fide* legal sanction reserved for this specific type of convicted offenders (3). If it is required that such a person, considering their mental state and personal circumstances, is admitted to a hospital for psychiatric care, the court will transfer this person to such a hospital, where the conditions include deprivation of physical liberty and coercive care interventions (4). A “*special discharge review*” (SDR) condition was added to these general criteria in 1992. The court can stipulate SDR if, as a result of the *mental disorder*,^2^ there is a risk that the person will relapse into crime of a severe nature (2, 4). SDR entails that all relaxations of liberty restrictions, e.g., outpatient treatment, conditional leave, and discharge, must be approved by an administrative court (5). Furthermore, if the forensic psychiatric care is to be continued, it has to be continued every six months by an administrative court, after application by the chief psychiatrist, regardless of whether the person has SDR as a supplement to their forensic psychiatric care or not. If no application to the court to continue the care has been made and the sentence does not include SDR, the patient is automatically discharged from the forensic psychiatric care. In addition, when the sentence includes SDR, the chief psychiatrist must also apply to the court for the patient to be discharged or transferred to outpatient care. An independent medical expert is to attend these hearings and the patient is entitled to a public defense counsel (2). Approximately 300 people are sentenced to forensic psychiatric care in Sweden each year, in comparison to approximately 12,000 who are sentenced to prison. About 80% of these 300 people also receive SDR as a supplement to their forensic psychiatric care (6).

A system of coercive detention in special mental healthcare facilities, subject to independent legal oversight review, is a common solution throughout the world, even if the legal procedures concerning mentally disordered offenders in Sweden is atypical in an international context. A systematic review of the empirical literature regarding decision-making in mental health tribunals found 50 papers, mostly from the United Kingdom, Australia and New Zealand but also from Canada and Ireland, that met the inclusion criteria. The researchers identified 11 different themes relating to shortcomings in current tribunal systems, e.g., tribunals are dominated by the health perspective, that the hearings function as “rubber stamps” for medical opinions, and that the decision is dominated around assessment of dangerousness (7). Pedersen et al. (8) have previously studied how patients experience mental health legal proceedings in terms of the therapeutic logic in the oral hearing. The interest for therapeutic logic emanates from previous studies in the Swedish context suggesting that patients are viewed as frail and volatile and how this creates a medical focus in court hearings that aims to reduce stress for the patient and not destroy the relationship between the treating psychiatrist and the patient (9). Pedersen et al. (8) tentatively raised the question whether the oral hearing in an administrative court might risk distressing the patient *because* of the courts’ very efforts to achieve the contrary.

The following attend these oral hearings; the patient, the patient’s counsel (optional), an external support person for the patient (optional), the chief physician or someone at the hospital who represents the chief physician, an independent medical expert, three lay judges and the presiding judge (10). The presiding judge also functions as a chairman and initiates the hearing by informing about which issues are to be considered. The lay judges and the presiding judge must familiarize themselves with the case and read the written application from the chief physician prior to the hearing. The chief physician has the floor first during the hearing, followed by the patient and his or her counsel. The counterparties then have the opportunity to put questions to each other, request clarifications etc. Finally, the independent medical expert has the floor and then the hearing is closed. The court’s decision in writing is usually sent to those concerned within the next couple of days (10).

Both staff and patients have reported patient participation being a challenge in forensic psychiatry in previous studies (11–16). One reason may be that the forensic psychiatric process is difficult to understand and is experienced as being slow and complex. The perception of participation seems to be linked to the experience of transparency and honesty in the care process (13). How these hearings in mental health review tribunals were perceived by patients, their family members and by professionals were investigated in a qualitative study from Canada. The different stakeholders found the patients to be treated respectfully and fairly during the hearings. However, the study also highlighted that patients often consider the length of time spent in forensic psychiatry as unfair and disproportionate to the crime, perceiving a sense of punitiveness (17).

The administrative court is such a core element in the forensic psychiatric care as it constitutes the legal authority that legitimizes the deprivation of liberty. It is often overlooked in research despite its obvious importance to the care process. Understanding this type of legal oversight review in the specific Swedish context can contribute to a greater understanding of these types of institutional solutions for managing the intersection of serious offending and serious mental illness. Studying the specific Swedish solution requires at the same time an understanding of the specific procedures involved in this context. These court proceedings are of fundamental importance for both the patient and for the conditions of the provided care. Studies aimed at a greater understanding of forensic psychiatric care and at improving the quality of this care should therefore focus on such aspects, including how patients subjectively experience the proceedings and their relationship to the care. To the best of our knowledge, there are no phenomenological studies from the patient’s perspective that focus on the lived experience of participating in the oral hearings in an administrative court.

## 2. Aim

The aim of this study was to describe patients’ lived experiences of participating in oral hearings in an administrative court concerning the continuation of their forensic psychiatric care.

## 3. Materials and methods

This is a phenomenological study, where the participants’ lived experiences of the phenomenon “participating in oral hearings in an administrative court concerning the continuation of forensic psychiatric care” is in focus. The interviews were conducted with a Reflective Lifeworld Research (RLR) approach (18). RLR has its foundation in the core theories of phenomenology, in particular Husserl’s lifeworld theory (19), the theory of intentionality (20), and later phenomenologists, e.g., Merleau-Ponty’s and Heidegger’s developments of the Husserlian phenomenology (18). Based on these theories and phenomenologists, Dahlberg et al. (18) designed a research approach and methodological principles for phenomenological research. Sundler et al. (21) have proposed a guidance for thematic analysis based on descriptive phenomenology, which has been applied in this study,

### 3.1. Settings

The current study was conducted at three forensic psychiatric clinics, on high security wards (level 2) and very high security wards (level 1) (22). A total of 12 different wards were included. A ward of this kind often houses 8–12 patients and has a high level of staff density. The administrative court had an onsite hearing room at each clinic. The presiding judge, independent medical expert, lay judges, and public defense counsel all come to the clinic once a week to participate in the hearings. Ward staff, usually licensed assistant mental health nurses, escort the patient to the administrative court. The staff that participate do not have an active role during the hearings but are responsible for the security and transport of the patient.

### 3.2. Informants

This study included 20 participants, all sentenced by a court to forensic psychiatric care with special discharge review. They were, at the time of the study, treated at a high or very high security level (levels 1 or 2) (22) at the time of the interview. Their length of care at the time of the interview varied from 1 to 32 years (mean = 6; median = 2,5), and the majority were men (*n* = 16 vs. *n* = 4 women). The age of the informants varied from 22 to 63 years (mean = 36; median = 33). A purposive sampling approach was applied to include a variety of participants in terms of age, length of care, and primary psychiatric diagnoses. Examples of psychiatric diagnoses were psychosis, autism spectrum disorders, and personality disorders.

### 3.3. Data collection

Data were collected in 20 lifeworld interviews (18). Eighteen interviews took place in a conversation room at the ward and two were conducted by telephone due to the COVID-19 pandemic. The first author conducted all the interviews, which lasted from 15 to 60 min and were audio-recorded and subsequently transcribed verbatim.

The treating psychiatrist assessed each patient’s mental state to ascertain whether or not he/she would be harmed by participating in an interview and whether they were fit or not to provide informed consent. This was performed prior to the patients being asked to participate in an interview. Ward liaisons then helped recruiting participants, providing information to patients and contacting the interviewer when patients showed interest in participating in the study. The first author gave additional information if somebody was interested and obtained written voluntarily informed consent. The information was given both in writing and orally and time was provided for questions to be asked and to make sure that the participants understood the purpose of the study and that the study did not have any impact on their care. The interviews were conducted using a reflective attitude, according to the principles of RLR (18). The initial question was: Can you tell me about the last time you participated in an administrative court for an oral hearing concerning the continuation of your forensic psychiatric care? Targeted questions were then asked, for example, How did you prepare for the hearings? Did you get any support from the staff? Was the public defense counsel a support for you? Follow-up questions were then asked, e.g.: Can you provide me with an example? How did it feel then?

### 3.4. Data analysis

The analysis is grounded in RLR (18) in terms of using a reflective and bridled attitude throughout the analysis, avoiding understanding the phenomenon too quickly. However, the structure of the analysis is a qualitative thematic analysis based on descriptive phenomenology according to Sundler et al. (21). This structure can be used for RLR, as well as for other phenomenological approaches. The goal of the thematic analysis is to understand patterns of meaning from lived experiences, in this case of participating in oral hearings concerning the continuation of forensic psychiatric care.

Firstly, the text was read several times with the intention of gaining a first tentative understanding of its meanings, and then re-read and meanings that corresponded to the study’s phenomenon were marked. These meanings were then compared to each other and clustered to gain a sense of patterns, where after the patterns were further examined. This process was characterized by a reflective, slow and open approach to the data; always by moving back and forth between the whole and its parts. Finally, the patterns were organized into themes, assisted by discussions between the researchers, aiming to outline inherent meanings from the participants’ lived experiences.

### 3.5. Ethical considerations

This study addressed a particularly vulnerable group of people and raised issues relating to their possibilities of providing informed consent given their mental state, the coercive nature of their care, and the integrity of personal information disclosed in the interviews. The study was approved by the Swedish Ethical Review Authority (Reg nr: 2019–02667) prior to its initiation. The interview material was stored so that no unauthorized person had access to it.

## 4. Results

The phenomenon is constituted by three themes that together form a whole.

### 4.1. A significant, correct but meaningless formality

The administrative court generates a contradictory experience as the value of it seems difficult to grasp. The hearings are described as functioning well in a formal way; the procedure is explained to be correct and fair from a legal perspective. However, the oral hearing is at the same time described as pointless if the physician applies for continued forensic psychiatric care. The hearing is not seen as a “real” legal proceeding where the outcome is open, but as a way to legitimize the continuation of the compulsory care, i.e., a formality.

The participants described it at the same time as being important for them to arrive at the court well-dressed and prepared to make a good impression in the hope of a positive outcome. They also report the oral hearing as being short, sometimes no longer than 5–10 min, and as stressful, as they anticipated a given, negative ending for themselves. On the one hand, the hearing is thus attributed a value and patients allow themselves to hope for a positive outcome, while on the other hand the overall description of the oral hearings is that they were characterized by the experience of the whole process being a window dressing, that it is in fact just a standard procedure that does not matter.

*‘I shower before, and then I put on some clothes I normally do not wear, jeans and a nice sweater. I think you should think about things like that. Or you should. The first impression says a lot’ (Participant 6).*

*‘No, but my experience is that it’s settled in advance when you go to the administrative court, it’s almost as though the judicial decision is already written’ (Participant 16).*

There is an experience that the oral hearings above all have the function of making the system appear legally secure. Being assigned a counsel is a form of guarantee that the procedure proceeds correctly from a judicial perspective. But the hearing is described as a “must” to participate in. It is described as having the function of legitimizing/protecting the physician:

‘*It’s not our rights they are protecting, the administrative court, it’s the physician’s rights.’ (Participant 1).*

The outcome of the oral hearing is perceived as predictable and that it is hopeless and “unnecessary” to participate in the administrative court if the physician has applied for continued care.

*‘Cannot I just stay in my room and get the decision, you think. It (the appeal) still says that the physician thinks that the care should be continued, so why go there.’ (Participant 20).*

The patients’ stories show how there are jokes at the wards about the administrative court and how new patients believe that the oral hearings matter. When patients come to forensic psychiatry, they first accentuate the value of what an oral hearing means for them, but then gradually realize that it takes time to get out. A patient with a long experience of forensic psychiatric care and participating in administrative court hearings describes how “new” patients act:

*‘They’ve got a counsel, they have nice clothes, they are very well prepared.. then they get up there and then it’s just …, the outcome in the administrative court is almost decided in advance, you could say. It goes without saying that they do not discharge a patient after 4 months who might be sitting for attempting to murder or something like that. Then you will not be discharged after 4 months. They go there very excited and like “yes, now I’m getting out” and then they come back here looking completely destroyed.’ (Participant 3).*

It is described as naive to believe that it is possible to be discharged at the administrative court unless discharge is part of a designated process. Instead, it is important to follow the pace and conditions of care, and not get too eager. The administrative court is thus an extension of the compulsory care and legitimizes the slow process. The physician can always find some reason why the length of stay should be continued.

*‘There are so many things that have to click in order for us.. us patients… if we are to be allowed to do anything. You know, we have to tick off half the bible, but if they want to release one then they can do as they please. So, all the power rests with them.. I can be perfectly healthy, I can have a low risk assessment.. If I do not have my own apartment. ‘Take it easy, you have to stay until WE find a place for you to live in, WE do it, when WE decide it to be appropriate’ (Participant 12).*

*‘I think they are quite harsh, you know. Because I sort of got a job. I have an accommodation, you know. But they do not take that in consideration so much you know. They listen more to what the physician says, and the physician usually says that you would reoffend, you know. I think it’s a bit strange, you know.’ (Participant 19).*

### 4.2. An imbalance of power within the hearings

The court hearings are characterized by a loss of power and being dependent on the physician’s goodwill. The counsel and the administrative court have a knowledge disadvantage compared to the psychiatrists. The two attending physicians have great power over the outcome of the negotiation and are considered to represent the “medical truth.” At the same time, the counsel is perceived as having limited influence. It is difficult for the counsel in relation to the physician to argue when the latter uses medical arguments. The judges and laymen also lack medical knowledge but are perceived to fully trust the physicians. The administrative court is described as being more interested in what the physician and the independent medical expert say. Many patients question the value of being assigned a counsel and wonder if they really can make an impact on the outcome.

*‘… it’s very difficult for a counsel to argue against a psychiatrist about medicine. And that’s what happens. My counsel sits and argues with someone who has five years at university in psychiatry, about psychiatry. About information she did not even receive in the first place’ (Participant 12).*

*‘How can you claim, as a severely mentally disturbed person who does not even serve a prison sentence and cannot spend time in jail? You have to be compulsorily medicated. How can anyone listen to you instead of the kind doctor who is so well educated and who is so talented, you know, an expert in forensic psychiatry, a specialist? How the hell are you going to get your voice heard and believed?’ (Participant 1).*

The system in the administrative court, to be assigned a counsel, to be given the possibility to make one’s voice heard is described as fulfilling. The counsel provides support and can present one’s case in a way that is suitable for a negotiation, and in cases where the patient is too nervous, the counsel can hold the entire speech.

*‘I get to say what I think, but then it’s formulated with more legal impact. My counsel has participated in many, many more oral hearings in the administrative court. She knows the physicians in a different way, I just come as myself and think what I think.’ (Participant 16).*

Replacing one’s counsel is described as bringing up negative emotions because it means having to explain and disclose oneself and one’s situation to a new person. Being given different counsels every time impairs the sense of continuity and creates frustration for the patient.

The physician is described as having the greatest influence on the outcome in the oral hearing. The staff at the ward may think differently from the physician, but that does not matter, according to the patients, who describe it is a frustration that the staff, who have no say, know them much better than the physician does, who only sees them once a week during the medical round.

*‘For starters, they control which medication you should have, they control which ward you should be in.. they even decide when and how you are ready to go out for walks with the staff. They decide when they think it is appropriate to apply for patient leave. They decide when it’s time for outpatient care, they decide on discharge. So, they kind of have power over the entire care process. That’s what they have.’ (Participant 5).*

The physician is described as having full control over the care process. If the physician has not applied for discharge, the care will be continued. There is a frustration that the nature or severity of the crime that led to the forensic psychiatric care does not seem to matter. If the physician wants the care to continue, it will do so even for a “minor” crime. Patients express this in different ways.

*‘It does not matter if you are convicted of murder or if you are convicted of theft, you get a new six-month lease (continued care). That’s how it is. People have been sitting here for 12 years for a bicycle theft. Others have served just as long for a serious crime. But everyone is judged the same when you are in the administrative court at an oral hearing because if the physician applies for continued care, then it is the physician’s opinion that matters, no one else. Not the crime, not the administrative court, nothing.’ (Participant 6).*

The patients describe a general mistrust of the independent medical expert’s capacity to form a proper opinion; the independent medical expert is perceived as always agreeing with the attending physician.

*‘Should the independent medical expert oppose the treating physician? That never happens.’(Participant 5).*

*‘The medical expert who is there makes a decision based mainly on the medical records. The expert, he does not meet people (patients) enough to be able to form a proper opinion’ (Participant 14).*

It is the forensic psychiatric care that controls which information the administrative court receives. Opposing the physician is described as risking that the physician needs to exaggerate in his or her application to outweigh the patient’s attempts to oppose. That can result in bad outcomes. It is described as better to play the game and just try to follow the process. The less you resist, the easier it is to get out.

### 4.3. Existential and practical disorientation

Participating in the administrative court can give rise to feelings of being exposed, especially when it is one’s dangerousness that is discussed during the oral hearings. It is a disorienting and lonely quest to find someone who can guide you through the system. The process is difficult to understand and gives few answers as to what is required to be discharged, which raises existential questions about how it is possible to get out of the compulsory care.

In addition, the oral hearings are a reminder of one’s deprivation of liberty and the reason for it. It is described as difficult to listen to when the administrative court describes one’s crime, it evokes painful feelings and memories.

*‘I do not want to rip open the wound, you know. For me, it’s hard enough to just go there and hear what they are saying.’ (Participant 10).*

What is dealt with in the oral hearing is how you are as a person. What matters is whether you are considered dangerous or unstable. It is not the deeds that made you come to the forensic psychiatric care but the person that is in focus.

*‘What you discuss is not what you have done. The court is discussing one’s humanity.. who you are as a person. That’s why it’s difficult, because in some way… what is being discussed is how unstable and dangerous you are’ (Participant 12).*

The experience is that, in the court, the focus is on the negative, the reasons to why the care should be continued, not on the progress that has been made. The emphasis is on risks. This is described as difficult for those who disagree. The physician’s application can be similarly experienced as harsh to read, especially if the portrayal of one’s person, in terms of dangerousness and so on, is perceived as being unfairly described. There is a desire to discuss the content in the application together with the staff or the physician. At the same time, it is difficult to talk to the staff about the physician’s application or about how it is in the administrative court, opinions about the medical expert etc. This is partly because it can end up in the medical records and partly because the staff cannot answer for the physician. Some patients experience it to be inconvenient to talk to the physician about it.

*‘It’s somehow so difficult to explain that the person whom one should have great confidence in, the physician, is also one’s counterpart. It’s difficult to know how to deal with that’ (Participant 16).*

The staff inform the patient about the date a couple of weeks before the oral hearing and ask if a public defense counsel is desired. There is then often a lack of more information and support. The process around the oral hearings is perceived as a supplementary process to the care process. Patients describe a desire to receive feedback on how things went in the administrative court both from the staff and from the judge, a desire to listen to how others than themselves perceived the hearing and their performance. On the other hand, staff are often described as not being able to be a support as they themselves lack experience and can thus not relate, they do not know how it is to be the subject of a negotiation in the administrative court.

*‘You know, during the time I’ve been here, you can say that it’s possible to do more than what’s already being done. During the whole process then absolutely, at least a little bit of support.. or just to feel that you can get some more information about these subjects, you know.’ (Participant 4).*

There are also descriptions that some in the staff try their best, both giving comfort and trying to be as supportive as possible, even if they are unable to explain the judicial points.

*‘I’m getting support.. I do.. the people who work here are great.. they are humane and professional in their way of working. They know how to deal with most things, you know. When we are sad, when we are angry.’ (Participant 20).*

The process around the oral hearings is in a way characterized by feelings of abandonment, that there is no one who shows commitment, no one to guide you through the system, and you are left fighting alone against feelings of frustration and powerlessness. It is difficult to understand the system and frustrating not to receive help from the staff to understand how the process works.

*‘They themselves say that “had it been up to me”… I’ve heard from many…"had it been up to me, I would have let you out from this place..” they say that themselves, “you know, that it’s the physician and the administrative court who decide.” They cannot do much for me more than what they are already doing in here…’(Participant 20).*

It is not sufficient to become well or be free of symptoms to be discharged from the forensic psychiatric care. The essential part is described by the patients as being one’s dangerousness. If a crime were to occur after discharge, a shadow would fall on both the physician and the administrative court.

*‘They do not want to stand there and explain why they let me out. It’s a hell of a risk for them. So, just for sake, it’s best if they lock him up for another six months’ (Participant 1).*

Patients describe how they feel like they are being tested in the administrative court, it is common with difficult questions or accusations that are not easy to answer. When the physician and the medical expert present their description of one’s behavior in the oral hearing, it is sometimes experienced as a trap where it is easy to be provoked. It is important in these situations not to lose one’s face or behave in a way that can be perceived as you are lacking an understanding of your own medical condition.

*‘If both the physician and the independent medical expert say that there is a severe mental disorder and that you have to stay at the clinic. Then that means that if I say “no, I’m not sick at all” you know, if I just insist on not having any problems. Then both the physician and everyone else immediately understands…that you are lacking ability to understand your medical condition, that you are not aware of your own problems’ (Participant 5).*

Being sentenced to forensic psychiatric care is described as a life sentence, it is difficult not knowing how long the care process will be. There is a wish that the administrative court had been more thorough in its assessment of the care. The experience of the administrative court making their decision primarily on the basis of the physician’s risk assessment makes the patient feel as though he/she is treated as a dangerous object.

## 5. Discussion

The results are presented in three themes: A significant, correct but meaningless formality; An imbalance of power within the hearings; and Existential and practical disorientation. The findings are linked to the question asked by many, for example Andreasson et al. (23), which is to what extent forensic psychiatric care should focus on curing and treating the patients’ health problems and to what extent it should be guided by protective obligations. They raised this question in the same study, where they showed that there was a weak relationship between the patients’ psychiatric needs and their length of care in Swedish forensic psychiatry. It is obvious in this study that this fact is difficult to comprehend for the patients, where the participants say, for example, “the oral hearing is unnecessary” “getting healthy is not enough,” and perceive that the staff are in agreement with them. The underlying origin of this is a longstanding issue in the ethics of forensic psychiatry about the ultimate goals of this practice, and how a complex goal structure leads to conflicting notions about the aim and value of the care (16, 24). This study confirms that this dilemma is vividly experienced also by the patients in relation to the administrative court hearings. There is no statistic about how often the court and the independent medical expert agree with treating psychiatrists regarding continuation of the forensic psychiatric care. That information would have served this study well.

Counsels and advocates have concerns about how well informed their client is about the proceedings in mental health review tribunals in research focusing on procedural fairness, but also about how it is difficult to challenge medical evidence efficiently and how the evidence relied upon is probative (25). Previous research, from different stakeholders involved in these kinds of proceedings, has also shown concerns, such as the tribunals being dominated by the medical perspective, functioning as rubber stamps for the care, and revolving around assessment of the patient’s dangerousness (7). The findings in the current study demonstrate how these potential shortfalls are also experienced in a very similar way by patients. For instance, the participants perceived that the counsel was powerless in relation to the psychiatrist’s medical statements or evidence, and that they found it difficult to comprehend why they must stay in compulsory inpatient care when not having a severe mental illness any longer. This study differs from others in that the patients describe their experience as something shared, well-known, and acknowledged by the staff.

Since these shortfalls and areas for improvement in mental health review tribunal proceedings are well-known and it seems as though the patients’ lived experiences of the oral hearings in Sweden are consistent with these descriptions, there are thus potentially negative consequences for the trust in forensic psychiatry care. If patients believe themselves to be in a system that not even the staff understand or agree with, this might lead to a resentment toward the care. We suggest that this, in turn, can lead to reduced compliance to the care, a decreased sense of agency, and fewer patient participation factors that we associate with quality in care.

The existential disorientation experienced by the patients is a particularly important aspect of our findings, as it is immediately linked to any ambition for forensic psychiatry ever succeeding with its patients. Patients describe their experience of hearings in administrative court as mainly being focused on risk and dangerousness, and that this predominates other factors they consider important, reinforcing an image of themselves as dangerous, and together with the perceived empty formality of the proceedings, as hopeless cases. This links to an observation by El-Alti and colleagues (16) that the complexity and rigidity of the care system can affect the care progression negatively, and that the court proceeding is a crucial arena for the patients’ experiences of care progression or its opposite. The patients can in this type of system view themselves as someone in continuous need of risk assessment and strict control. It is necessary to avoid further stigmatization of the patients, so that they are not trapped in a notion of themselves as “uncurable cases” that will always be dangerous, while also being transparent in terms of assessed risks.

The concept of dangerousness requires a specific elaboration, since one of the outspoken goals of forensic psychiatry is societal protection through risk management of people with severe mental disorders and increased risk of recidivism into severe crime. There is substantial evidence that people with severe mental disorders, e.g., schizophrenia and bipolar disorder, in general pose a greater risk of violent acts than people without severe mental disorders, even when accounting for risk increase by comorbid substance use [see, e.g., (26–28)]. Prisoners in correctional institutions diagnosed with psychiatric disorders have an increased risk for violent reoffending. The risk increases stepwise with the number of psychiatric disorders, and it is thus relevant to give treatment to those suffering from mental disorders in the prison services (29). However, Buchanan and Grounds (30) suggested that psychiatric disease is treated in psychiatric care, while risks may arise independently of such a disease—even if it is taken into account that several mental health conditions include norm-breaching behavior as diagnostic criteria (31). Thus, if the aim of forensic psychiatry is set as the minimizing of the risk of reoffending in general, the care may succeed in psychiatric terms but still be bound to fail in terms of risk prevention. Vivid experiences of precisely this dilemma have been described in a recent study of Swedish forensic psychiatric care (16). Buchanan and Grounds (30) argue that this situation is likely to arise in a social and political environment that emphasizes crime prevention as a major and dominant value.

Correctional services make a risk assessment of a person sentenced to prison in Sweden, but this is just a matter of security classification and is not related to the length of stay. The only exception is if lifetime inmates apply to have their sentences time-limited (32). Forensic psychiatry, on the other hand, works very actively with risk assessments (33, 34), and it has been demonstrated that the perceived risk is in itself a predictor for length of stay (23). However, many risk factors for violence are not linked to severe mental disorders. It is known for example that a high score on the Historical Clinical Risk-20 (HCR-20) (35) is negatively associated with discharge (36). Personality disorders, especially antisocial personality disorder, and substance use disorders increase the risk of reoffending (37, 38), and a history (and early onset of) violence has repeatedly been demonstrated as the strongest risk factor for violent recidivism [e.g., (39, 40)]. There is evidence that specific psychotic symptoms (e.g., hallucinations and delusions, especially of a paranoid/persecutory character) are linked to an increased risk for violence in people with severe mental disorders (41, 42). However, there is literature suggesting that many crimes committed by offenders with severe mental disorders are not motivated by symptoms of, e.g., psychosis and that it is difficult to distinguish between symptoms specific to major mental disorders and features that can be found in the group of offenders without mental illness (43). General risk factors, e.g., antisocial traits, low self-control, stimulation seeking, and an established criminal history seem to predict recidivism alone, while factors unique to mental illness have no incremental utility (31, 44).

Personality disorders and substance use disorders are of course psychiatric disorders but not disorders severe enough to sentence someone to forensic psychiatry, that the individual also suffers from some kind of psychotic condition is also required (1). However, it is not unusual that patients in forensic psychiatry have personality disorders and substance use disorders in combination with a psychotic condition (45). Forensic psychiatric patients might be very similar to prisoners after being treated for the psychotic condition, but then they are in a system where risk and dangerousness matter, unlike those with the same conditions in prison.

Forensic psychiatric care does not exist in a vacuum; patients compare the care with a comparable prison sentence. A clinical implication from this study is that when explaining how forensic psychiatric care works, it is important not to simplify the explanation as a question about getting well from a psychotic condition that counts as a severe mental illness. It is important to be transparent that the risk perspective factor might be the most important in terms of length of stay. Furthermore, involving the patient actively in collaborative violence risk management could potentially increase the patient’s own understanding of how to progress in the care, while considering risks.

The current findings show how patients describe a desire to receive feedback on their experiences in the administrative court, and to be guided for future hearings. Patients describe good examples of staff being supportive and comforting. However, since the oral hearing is described as something separate from the care, staff often lack own experience or knowledge of the process concerning the continuation of the care in an administrative court. Managers in the care system thus need to ascertain that staff are well informed of the judicial context for forensic psychiatric care, so that the staff can answer patients’ questions appropriately, and not unwittingly mislead them.

The existential disorientation portrayed in the current study needs to be addressed, and that entails a change from the practicalities of the care to the very basis of providing care. A plethora of nursing and caring studies emphasizes the importance of establishing significant and caring relationships with patients, through a caring approach from staff (46, 47). There is also a well-known complexity of dealing with the dual tasks, both caring for the patient and protecting the society from the same patient, which is often referred to as the dual role dilemma (24). The part of that dilemma focusing of social protection, i.e., risk and dangerousness, is very prominent in the oral hearings concerning the continuation of the patient’s forensic psychiatric care. Discussions about oral hearings might in a way touch the most essential core of the complexity in forensic psychiatric care, it is hard to discuss the outcome from the oral hearings without considering the reasons for why the patient will remain in the care and what must be accomplished to be discharged. This is a difficult task from a staff perspective, especially since the goal for forensic psychiatric care is unclear (16).

The experience of care quality, patient participation, and good and significant relationships is very much associated with an experience of being listened to and getting fair and honest explanations about the decisions concerning one’s care (13). The current results provide strong reasons for emphasizing the importance of carers adopting a caring approach and a patient perspective, something which is embedded in nursing studies performed in this kind of environment (46, 48–50). It is of value just to take the opportunity to sit down and listen to the patient’s frustration even if it is difficult to explain the forensic psychiatric system and its jurisprudence. It takes courage to be present in the encounters with patients in their suffering, and it is indeed challenging to be in a position of not knowing how to approach patients in a way that helps them understand themselves from a place of respect and restored dignity. Furthermore, it does not just concern being confronted with the patient’s suffering, the care is also confronted with his/her own reaction to that patient’s suffering (51). We thus suggest once more that avoiding these kinds of encounters or discussions on forensic psychiatric care risks enhancing the suffering from a patient perspective.

## 6. Methodological reflections

Patients in forensic psychiatry consist of a very heterogeneous group (45, 52). This is reflected in the group of patients participating in this study, who present a rich variation of individual characteristics, including differing psychiatric disorders. It is important to have a rich variation of participants in RLR (e.g., gender, age, length of stay) and to not exclude a certain group (18). Some participants in the current study were very eloquent and high-functioning, while others were more affected by their disorders. This variation contributed to some of the interviews being shorter and others longer.

Phenomenology has been prominent in nursing studies for decades. Its progress has made an important contribution to the practice of psychiatry and understanding of psychopathology. Phenomenology has not only made a difference in the understanding of data but also in how data are collected in the first place, with special interview techniques etc. Phenomenology is known for its features that are important for qualitative research, e.g., its criticism of scientism and ambition to develop an open-minded attitude. Above all, its acknowledgment of the importance of the lifeworld (53). Allowing the participants to take the expert role of the phenomenon “participating in oral hearings in an administrative court concerning their forensic psychiatric care” through their own lived experience is a beneficial method for not steering the interviewee. It is obvious that the findings in the present study are very similar to those in previous research with other stakeholders than patients and this has been achieved without using a battery of questions, which we know from the literature and previous research has been discussed as problematic or a challenge in these types of proceedings concerning psychiatric care. However, in hindsight, it would have been interesting to ask questions more specifically focusing on the degree to which patients have been given explanations about the concept of severe mental disorder and the reason for why they are not discharged. For example, if it has been stated that they do not longer present a severe mental disorder but are still considered to be dangerous.

There is a significant difference between trying to understand the complexity of meanings in the data rather than measuring the frequency of every assertion. This might contribute to phenomenological analysis appearing more abstract in comparison to other analyses. A great deal of the process is to use, and understand, the ontological and epistemological foundations of phenomenology and its methodological principles, e.g., emphasizing openness, questioning pre-understanding, and adopting a reflective attitude (21). A more general structure of meanings is often presented as an essence in some phenomenological analyses (18, 54), even though thematic analysis occurs from time to time. We chose to present a thematic analysis that organized patterns of meaning into themes based on the work of Sundler et al. (21). We maintain that the phenomenon is of such a character that it would not be purposive to present the results on an essential level. Sundler et al. (21) asserted that meaning-oriented themes contribute to robust qualitative research findings, but that it is important to present the findings both as descriptive text-based on lived experience and as concrete expressions. We maintain that it would not add anything to the reader to present the results on an essential level (as an essence), and we also believe that this way of presenting the results can be more reader-friendly for those not so familiar with phenomenological research.

## 7. Conclusion

Participating in an oral hearing in an administrative court concerning the continuation of forensic psychiatric care is often experienced as challenging by the patients. This is partly due to the forensic psychiatric care structure and that the purpose of the hearings is difficult to comprehend and is perceived as unjust by patients. Another challenge is of a more existential dimension; as a patient being the main character in a hearing is probably a situation that would have caused stress for anyone. However, the focus on dangerousness can make this experience even more intense. If acknowledging that this part of the forensic psychiatric care is experienced by patients as difficult to understand, unjust and sometimes even agonizing, we believe that the forensic psychiatric services can do much more to make the care more understandable for patients. However, this requires that the staff themselves understand the purpose of the administrative court hearings and, further, have an understanding for the goals of forensic psychiatric care. Finally, the results imply that patients feel they are left alone with their thoughts and questions. Creating opportunities for staff to sit down and talk about the patients’ experiences of participating in oral hearings, both as a preparation prior to a hearing and an evaluation after a hearing, could thus form a basis for an increased common understanding between patients and staff that might subsequently benefit the care process for the patient.

## Data availability statement

The datasets presented in this article are not readily available because it is transcribed interviews, even if they are anonymous. The ethics do not allow sharing to others than the researcher within the project. Requests to access the datasets should be directed to andreas.soderberg@lnu.se.

## Ethics statement

The studies involving human participants were reviewed and approved by Swedish Ethical Review Authority. The patients/participants provided their written informed consent to participate in this study.

## Author contributions

AS contributed with the planning of the study, performed all interviews, led the analysis, and wrote the manuscript. MW made significant contribution in the text and helped with the planning of the project. CM made significant contribution in the text. MR gave comments on the manuscript. UH supervised and helped planning the whole research project and commented and shaped the manuscript. All authors contributed to the article and approved the submitted version.

## Funding

This work was supported by the Swedish Research Council for Health, Working Life and Welfare under Grant no. 2018-01409, Medical Research Council of Southeast Sweden under grant no. 2019-930801, Department of Research and Development, Region Kronoberg, 2019-933814, 2019-930775, and supported by the Regional Forensic Psychiatric Clinic in Växjö.

## Conflict of interest

The authors declare that the research was conducted in the absence of any commercial or financial relationships that could be construed as a potential conflict of interest.

## Publisher’s note

All claims expressed in this article are solely those of the authors and do not necessarily represent those of their affiliated organizations, or those of the publisher, the editors and the reviewers. Any product that may be evaluated in this article, or claim that may be made by its manufacturer, is not guaranteed or endorsed by the publisher.

## Footnotes

^1^Severe mental disorder is a legal concept; Conditions where the person has a disturbed perception of reality. For example, psychosis or severe depression with suicidal thoughts or plans. Or severe personality disorders or anxiety disorders (with severe compulsiveness or inability to control one’s impulses) which gives a disturbed perception of reality. Or is in such a serious mental crisis that the level of mental functioning is affected as to be of a psychotic nature (1).

^2^Note that the used term in this section is mental disorder, not severe mental disorder. The law does not stipulate that the patient must still have a severe mental disorder, when the patient has SDR as a supplement to their forensic psychiatric care, as long the patient has a risk of relapsing in severe criminality the care must be continued.
